# Extracellular PPM1A promotes mineralization of osteoblasts differentiation in ankylosing spondylitis via the FOXO1A‐RUNX2 pathway

**DOI:** 10.1111/jcmm.17685

**Published:** 2023-02-09

**Authors:** Subin Weon, Sungsin Jo, Bora Nam, Sung Hoon Choi, Ye‐Soo Park, Yong‐Gil Kim, Tae‐Hwan Kim

**Affiliations:** ^1^ Hanyang University Institute for Rheumatology Research (HYIRR) Seoul Korea; ^2^ Department of Translational Medicine, Graduate School of Biomedical Science and Engineering Hanyang University Seoul Korea; ^3^ Department of Rheumatology Hanyang University Hospital for Rheumatic Disease Seoul Korea; ^4^ Department of Orthopedic Surgery Hanyang University Seoul Hospital Seoul Korea; ^5^ Department of Orthopedic Surgery Guri Hospital, Hanyang University College of Medicine Guri Korea; ^6^ Division of Rheumatology, Department of Medicine University of Ulsan College of Medicine, Asan Medical Center Seoul Korea

**Keywords:** ankylosing spondylitis (AS), forkhead box O1A (FOXO1A), osteoblasts, protein phosphatase magnesium‐dependent 1A (PPM1A), runt‐related transcription factor 2 (RUNX2)

## Abstract

Protein phosphatase magnesium‐dependent 1A (PPM1A), serine/threonine protein phosphatase, in sera level was increased in patients with ankylosing spondylitis (AS). Preosteoblasts were differentiated actively to matured osteoblasts by intracellular PPM1A overexpression. However, it was unclear whether extracellular PPM1A contributes to the excessive bone‐forming activity in AS. Here, we confirmed that PPM1A and runt‐related transcription factor 2 (RUNX2) were increased in facet joints of AS. During osteoblasts differentiation, exogenous PPM1A treatment showed increased matrix mineralization in AS‐osteoprogenitor cells accompanied by induction of RUNX2 and factor forkhead box O1A (FOXO1A) protein expressions. Moreover, upon growth condition, exogenous PPM1A treatment showed an increase in RUNX2 and FOXO1A protein expression and a decrease in phosphorylation at ser256 of FOXO1A protein in AS‐osteoprogenitor cells, and positively regulated promoter activity of RUNX2 protein‐binding motif. Mechanically, exogenous PPM1A treatment induced the dephosphorylation of transcription factor FOXO1A protein and translocation of FOXO1A protein into the nucleus for RUNX2 upregulation. Taken together, our results suggest that high PPM1A concentration promotes matrix mineralization in AS via the FOXO1A‐RUNX2 pathway.

## 1 INTRODUCTION

Ankylosing spondylitis (AS) is chronic inflammatory arthritis characterized by excessive bone‐forming activity, syndesmophyte formation and progression of spinal ankylosis.^1^, ^2^, ^3^, ^4^, ^5^ Clinically, there are therapeutic drugs targeting TNF and IL17 in patients with AS, but these drugs do not prevent spinal ankylosis. In addition, little is known that the exact pathological mechanism for excessive bone formation in AS.^6^, ^7^, ^8^, ^9^

Protein phosphatase magnesium‐dependent 1A (PPM1A), a member of the protein phosphatase 2C family of serine/threonine phosphatases, is known to dephosphorylate target proteins such as AMP‐activated protein kinase (AMPK), p38, JNK and Smad1/2/3.^10^, ^11^, ^12^, ^13^, ^14^, ^15^ The previous studies have shown the elevated level of anti‐PPM1A autoantibodies was positively correlated with AS disease activity and associated with a high grade of sacroiliitis.^16^ Macrophage‐specific PPM1A deficiency mice showed increased osteoclast differentiation by inducing RANK expression.^17^ Moreover, in AS patients treated with TNF inhibitor, a high level of anti‐PPM1A autoantibodies was a risk factor for radiographic progression and in vitro, exogenous PPM1A treatment increased matrix mineralization of primary osteoprogenitor during osteoblast differentiation.^18^ These findings suggest that PPM1A may contribute to dynamic bone metabolism and formation in AS leading to spinal ankylosis.

Forkhead box O (FOXO) plays a crucial role in process of bone formation.^19^, ^20^ Especially, FOXO1A is mainly involved in osteogenesis and bone development; depletion of FOXO1A has been reported in a decrease in osteoblast proliferation, bone formation and bone volume.^21^ Although there is still controversy in the literature as to the regulatory mechanisms in FOXO1A and RUNX2 expressions. Some studies have demonstrated that FOXO1A can positively regulate RUNX2 transcript, whereas other studies have opposite findings.^22^, ^23^, ^24^ It is thought that FOXO1A and RUNX2 play a crucial role in bone development and formation by coordinating with each other.

Although exogenous PPM1A treatment induced matrix mineralization in AS‐osteoprogenitor cells was previously reported,^18^ the regulatory mechanism remains to be established. In this study, we investigate the underlying molecular mechanism of PPM1A‐induced matrix mineralization of AS‐osteoprogenitor cells during osteoblast differentiation. We, therefore, propose the novel mechanism that PPM1A positively regulates RUNX2 expression by inducing dephosphorylation of FOXO1A protein.

## 2 MATERIALS AND METHODS

### 2.1 Human facet joint tissue

We obtained facet joints from seven AS patients who met the modified New York criteria^25^ and from seven patients with non‐inflammatory disease as the disease controls. This study was conducted in according with the Helsinki Declaration and approved by the Institutional Review Board of Hanyang University Hospital (Seoul: 2014‐05‐002 and Guri: 2014‐05‐001).

### 2.2 Immunohistochemistry (IHC)

IHC procedures were previously reported.^26^ Facet joint tissues were fixed with 10% formalin for 3 days, decalcified by 10% formic acid for one week and embedded in paraffin. The paraffin blocks were cut into 5–7 mm thick sections. Briefly, tissue slides were deparaffinized, dehydrated, incubated with Antigen Retrieval Kit (VitroVivo Biotech, VB‐6009), permeabilized with 0.3% Triton X‐100 in 1× TBS‐T and eliminated endogenous peroxidase with BLOXALL (Vector Lab, SP‐6000). Tissue slides were then followed by incubation for overnight at 4°C with the appropriate primary antibodies in antibody diluent (DAKO, S3022), ABC kit components (Vector Lab, PK‐6102), DAB substrate kit (Vector Lab, sk4100), counterstaining with haematoxylin (Merck, 1.05174.0500) and mounting with Permanent mounting medium (Vector Lab, H‐5000). Images were collected with a Nikon eclipse Ti‐U microscope. Five fields for each sample were acquired randomly at 200× magnification.

### 2.3 Isolation of osteoprogenitor cells and osteoblast differentiation

The protocols for isolation of osteoprogenitor cells were previously reported.^26^, ^27^, ^28^, ^29^, ^30^, ^31^ Surgical bone were split into pieces using rongeur, followed by washed with 1× PBS supplemented with 1% penicillin and streptomycin (Gibco, 15140122) to remove all marrow cells and blood. The bone tissue were cultured in DMEM‐HG (Hyclone, SH30243.01) supplemented with 10% FBS (Gibco, 16000044) and 1% penicillin and streptomycin (Gibco, 15140122) at 37°C in 5% CO_2_ conditioned incubators. Primary osteoprogenitor cells obtained from passage 1 to 5 were used in all subsequent cell experiments. To identify the effect of PPM1A (YBDYbiotech, REC120) on osteoblast differentiation, AS‐osteoprogenitor cells were seeded in a 96‐well plate at 1E4 cells/well and incubated for 24 h. The cells were stimulated with osteogenic medium containing 50 μM ascorbic acid (AA; Sigma, A4544), 10 mM β‐glycerophosphate (β‐GP; Santa Cruz Biotechnology, sc‐220452A) and 100 nM dexamethasone (Dex; Sigma, D2915) to induce osteoblast differentiation. Differentiation medium was changed every 3–4 days.

### 2.4 Assessment of osteoblast differentiation

The matrix maturation of osteoblast differentiation was assessed by alkaline phosphatase (ALP; Sigma, 85 L2) and collagen using Picro‐Sirius Red (COL; Abcam, ab150681) staining. The matrix mineralization of osteoblast differentiation was assessed by Alizarin red (ARS; Sigma, A5533), hydroxyapatite (HA; Lonza, PA‐1503) and Von kossa using 1% silver nitrate solution (VON; Sigma, S7179) staining. After staining, the wells were imaged by a Nikon eclipse Ti‐U microscope (Nikon, MEA510AA). For ARS staining quantification, stained well were incubated with 200 μL of 10% acetic acid at 37°C for 2 h. Extracted solution was added 80 μL each well on new 96‐well plate and measured at 450 nm with a multi‐plate reader (Thermo Fisher, 51119000). For HA staining quantification, stained well were measured at an excitation wavelength of 492 nm and an emission wavelength of 550 nm with a multi‐plate reader. For VON staining quantification, area of stained well images was analysed by Image J.

### 2.5 Immunofluorescence (IF)

Ankylosing spondylitis ‐osteoprogenitor cells were fixed with 4% paraformaldehyde (PFA; FUJIFILM, 163‐20145) at RT for 15 min, blocked with 10% serum for 1 h and incubated with primary antibody at 4°C overnight. The cells were stained with Alexa 488‐conjugated Goat Anti‐mouse IgG (Invitrogen, A‐11001) and Cy3‐conjugated Goat Anti‐Rabbit IgG secondary antibody (Jackson Immunoresearch, 111‐165‐144) counterstained with DAPI (VECTASHIELD, H‐1200) for 1 h. Each slide was observed in high‐power fields (HPF) at 400x magnification. IF images were captured using LAS version 4.2.1 software with a confocal microscope (Leica Microsystems GmbH, TCS SP5). Positive cells for each specimen were randomly visualized without non‐specific signals.

### 2.6 Real Time‐qPCR (RT‐qPCR)

Total mRNA of the AS‐osteoprogenitor cells was extracted using TRIzol® reagent (Invitrogen, 15,596,018), according to the manufacture's instruction. cDNA was synthesized using 1 μg mRNA and the cDNA synthesis kit (Thermo Fisher, K1622). The cDNA synthesis conditions were as follows: 42°C for 1 h and then 70°C for 10 min. RT‐qPCR was performed through a CFX96 RT‐qPCR detection system (Bio‐Rad Laboratories, BR18B‐5200) following the manufacture's instruction. The thermocycling condition as follows: Initial denaturation at 95°C for 3 min, followed by 50 cycles of 95°C for 10 s, 60°C for 30 s and 95°C for 10 s. The expression of each target gene was normalized to that of human GAPDH. The primers used for RT‐qPCR are described in Table S1.

### 2.7 Immunoblotting assay (IB)

For the immunoblotting assay, the stimulated cells were washed with 1× PBS and lysed in 1× RIPA buffer (50 mM Tris–HCl pH 8.0, 150 mM NaCl, 0.1% SDS, 0.6% Na‐deoxycholate, 1% Triton X‐100) containing protease inhibitors (Calbiochem, 535140) and phosphatase inhibitors (Cell Signalling, 5870 S). Lysed samples were incubated on ice for 15 min followed by centrifugation at 12,000× *g* for 15 min at 4°C. The protein lysates were determined using a Bradford protein assay (Bio‐Rad Laboratories, 5000006). Protein samples (25 ~ 50 μg) were separated by SDS‐PAGE and then transferred to nitrocellulose membranes (GE Healthcare, 10600002). The membranes were blocked with 5% non‐fat milk in TBS with 0.1% Tween‐20 at RT for 1 h, followed by incubation with specific primary antibodies at 4°C overnight. Then, the membranes were incubated with secondary antibodies at RT for 1 h. The membranes were visualized with Pierce ECL kits (Themo Fisher, 34580) and collected by chemiluminescence imaging system (Uvitech System). The primary and secondary antibodies used for the immunoblotting assay are described in Table S2.

### 2.8 Promoter activity assay

SaOS2 cells were seeded in a 60 mm plate at 2E5 cells/well and co‐transfected with ALP, OSE or OCN promoter (3 μg/well) and renilla (0.3 μg/well) using Lipofectamine® 3000 (Thermo Fisher, L3000015). One day after transfection, the cells were reseeded in a 12‐well plate at 5E4 cells/well and incubated for a day. On the following day, the cells were treated with PPM1A for 24 h and lysed to measure promoter activity by the Dual‐Luciferase Reporter Assay system (Promega Corporation, E1500) according to the manufacturer's instructions. The promoter activity data were normalized to the renilla values.

### 2.9 Nucleus/cytoplasm fractionation

Ankylosing spondylitis ‐osteoprogenitor cells were stimulated with PPM1A for 24 h. Cell pellets were lysed by tapping with 1× cytoplasm buffer (0.2% Triton X‐100, 200 mM Tris–HCl pH 8.0) containing protease inhibitors and phosphatase inhibitors. The lysates were incubated on ice for 10 min and centrifugated at 848 *g* for 10 min at 4°C. The supernatants were collected for cytoplasm proteins. Afterward, the pellets were washed with 1X cytoplasm buffer twice, and the pellets were lysed by pipetting with 1X nucleus buffer (1% Triton X‐100, 200 mM Trix‐HCl pH 8.0, 400 mM NaCl) containing protease inhibitors and phosphatase inhibitor. The lysates were incubated on ice for 10 min, followed by centrifugation at 18473 *g* for 15 min at 4°C. The supernatants were collected for nucleus proteins. Collected cytoplasm/nucleus proteins were subjected to immunoblotting.

### 2.10 Statistical analysis

Graph‐Pad Prism 7.0 was used for statistical analysis and images. Two unpaired and paired groups were compared by using Mann–Whitney U test (nonparametric variables) and Wilcoxon test (parametric variables), respectively. When multiple groups were compared, two‐way ANOVA followed by Tukey's multiple comparison test was used. The data are expressed as the standard error of the mean (SEM) (*n* ≥ 3). The asterisks represent the level of statistical significance (*p < 0.05, **p < 0.01, ***p < 0.001).

## 3 RESULTS

### 3.1 PPM1A and RUNX2 were increased in AS facet joint

**FIGURE 1:**
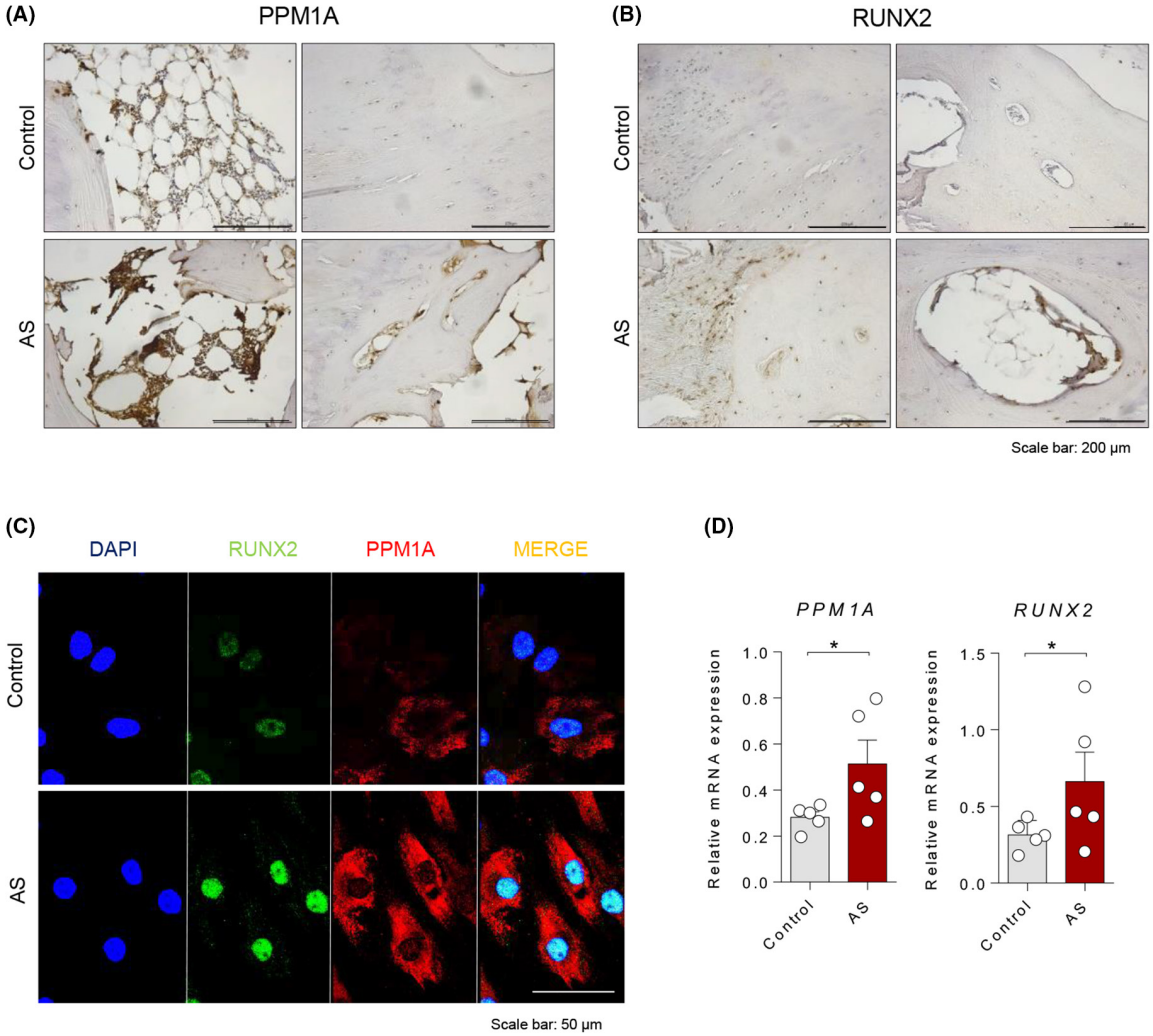
PPM1A and RUNX2 were increased in AS facet joint. (A) PPM1A and (B) RUNX2 in facet joints tissue of AS or disease control were stained by immunohistochemistry. Scale bar is 200 μm. (C) Control or AS‐osteoprogenitor cells were treated with PPM1A for 24 h and stained with anti‐RUNX2 (green), anti‐PPM1A (red) and DAPI (blue). Scale bar is 50 μm. (D) mRNA basal level for PPM1A and RUNX2 was verified by RT‐qPCR (*n* = 5 per each group). *p < 0.05.

We observed that PPM1A and RUXN2 expressions were high in bone marrow and bone‐lining cells of AS facet joint compared to control, as revealed by immunohistochemistry (Figure 1A,B). PPM1A protein in the cytoplasm and RUNX2 protein in nucleus were observed and those expressions were relatively greater in AS‐osteoprogenitor cells than in control (Figure 1C). Also, mRNA expression of PPM1A and RUNX2 were significantly elevated in AS (Figure 1D). These results suggest that expressions of PPM1A and RUXN2 are increased in AS facet joint.

### 3.2 Exogenous PPM1A induced matrix mineralization by increases in RUNX2 and FOXO1A expressions

To determine the involvement of PPM1A in regulating human osteoblasts differentiation, osteoprogenitor cells were differentiated into osteoblasts until 21 days. During differentiation, matrix maturation (alkaline phosphatase; ALP and type 1 collagen; COL) and matrix mineralization (Alizarin Red S; ARS, Von Kossa; VON, Hydroxyapatite; HA, and Bright field; BF) were gradually increased at each time point (Figure S1A). Interestingly, immunoblotting showed an increase in both PPM1A and RUNX2 expressions until 14 days during differentiation (Figure S1B). These data suggest that PPM1A is upregulated in the osteoblasts differentiation of osteoprogenitor cells.

**FIGURE 2:**
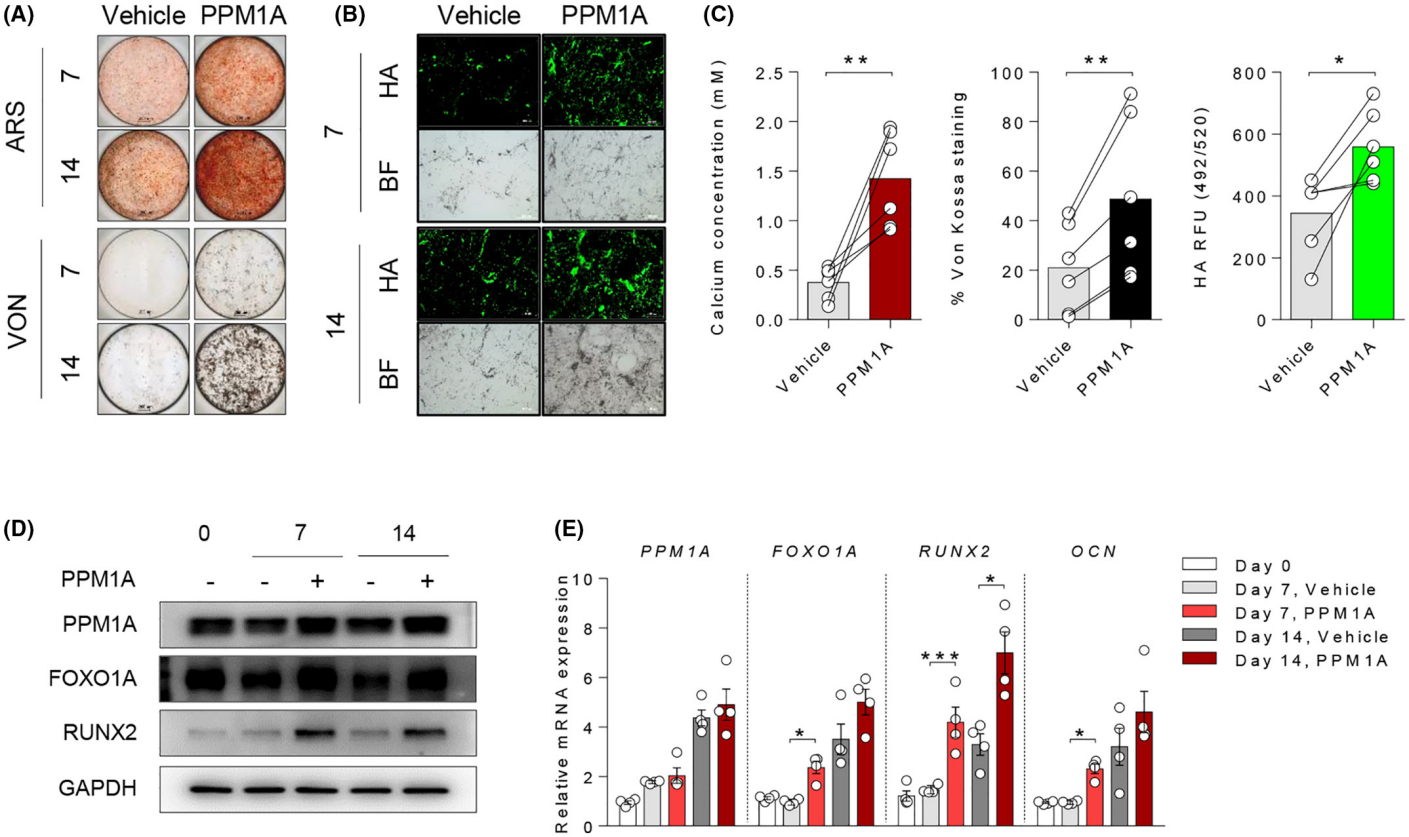
Exogenous PPM1A induced matrix mineralization by increases in RUNX2 and FOXO1A expressions. AS‐osteoprogenitor cells were differentiated with osteogenic medium in the presence of vehicle or PPM1A. Analysis of (A) ARS and VON and (B) HA staining as indicated days. Representative images are shown. Scale bar is 200 μm. (C) Quantitation of ARS, VON and HA staining at 14 days (*n* = 6 per each group). Differentiated cells were analysed by (D) Immunoblotting assay for protein level and (E) RT‐qPCR for mRNA level as indicated days (*n* = 4 per each group). *p < 0.05, **p < 0.01, ***p < 0.0001.

We previously reported that exogenous PPM1A treatment promotes matrix mineralization in AS‐osteoprogenitor cells.^18^ In consistent with the previous reports, exogenous PPM1A treatment significantly enhanced matrix mineralization of AS‐osteoprogenitor cells (Figure 2A‐C). Notably, the mRNA and protein levels of RUNX2 were significantly increased in the exogenous PPM1A treatment group compared to the vehicle group (Figure 2D,E). OCN as matrix mineralization marker of bone and FOXO1A expressions were both augmented by exogenous PPM1A treatment during differentiation. These results suggest that PPM1A promotes matrix mineralization in AS‐osteoprogenitor cells accompanied by upregulating FOXO1A and RUNX2 expressions.

### 3.3 Exogenous PPM1A treatment increases RUNX2 expression by inducing dephosphorylation of FOXO1A protein

**FIGURE 3:**
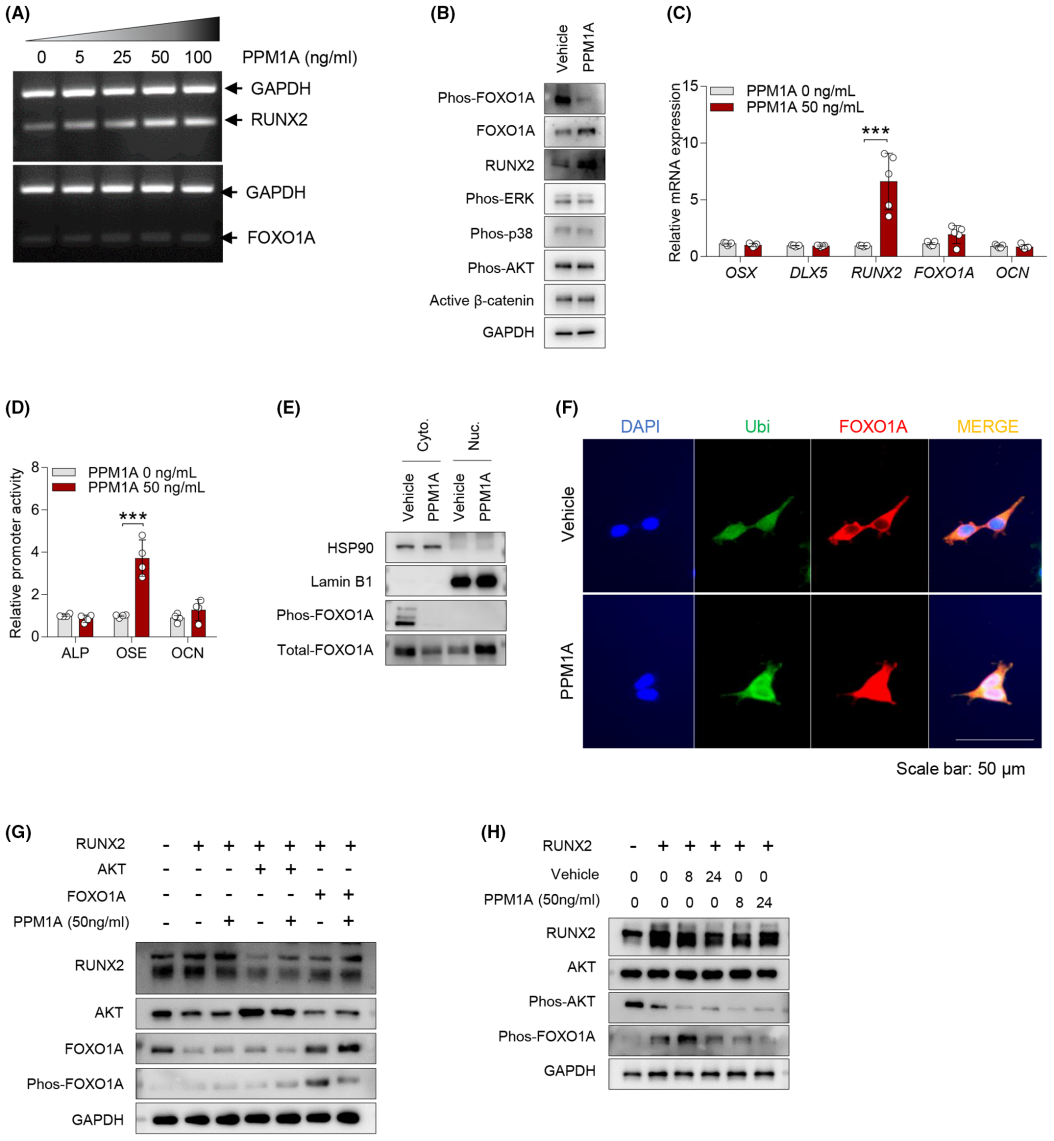
Exogenous PPM1A treatment increases RUNX2 expression by inducing dephosphorylation of FOXO1A protein. (A) AS‐osteoprogenitor cells were treated with various PPM1A doses for 24 h and analysed by RT‐PCR. AS‐osteoprogenitor cells were treated 50 ng/mL PPM1A for 24 h and analysed by (B) immunoblotting and (C) RT‐qPCR (*n* = 5 per each group). (D) SaOS2 cells were transfected with ALP, OSE or OCN promoter plasmids, followed by treatment with PPM1A for 24 h and then analysed by luciferase assay (*n* = 4 per each group). (E) AS‐osteoprogenitor cells were fractionated cytoplasm and nucleus protein after PPM1A stimulation for 24 h. The cytoplasm and nucleus proteins were analysed by immunoblotting. (F) AS‐osteoprogenitor cells were treated with PPM1A for 24 h and stained with anti‐Ubi (green), anti‐FOXO1A (red) and DAPI (blue). Scale bar is 50 μm. (G) Control‐osteoprogenitor cells were transfected with RUNX2, AKT or FOXO1A as indicated and treated with vehicle or PPM1A for 24 h. Proteins were analysed by immunoblotting. (H) Control‐osteoprogenitor cells were transfected with RUNX2 and treated with vehicle or PPM1A as indicated times. Protein levels were analysed by immunoblotting. ***p < 0.0001.

We next investigated the regulatory mechanism of exogenous PPM1A treatment‐induced FOXO1A‐RUNX2 expressions. Both mRNA and protein expressions of RUNX2 were elevated in various exogenous PPM1A treatments, while the treatment induced upregulation of FOXO1A protein without the transcript change (Figure 3A). Exogenous PPM1A treatment showed no alteration in phosphorylation of ERK, p38, AKT, and active b‐catenin proteins in AS‐osteoprogenitor cells, but a decrease in phosphorylation at serine 256 of FOXO1A protein (Figure 3B). Changes in exogenous PPM1A treatment‐mediated FOXO1A and RUNX2 transcript levels were varied by RT‐qPCR (Figure 3C). Moreover, exogenous PPM1A treatment especially upregulated activity of OSE promoter, RUNX2 protein‐binding motif, but not in ALP and OCN (Figure 3D). Exogenous PPM1A treatment showed a reduction in phosphorylated and total cytoplasmic FOXO1A protein and accumulation of total FOXO1A protein (Figure 3 E). As shown in Figure 3F, the translocation of cytoplasmic FOXO1A protein into the nucleus was induced by exogenous PPM1A treatment. Furthermore, since FOXO1A protein has been reported to be negatively regulated by serum‐induced AKT,^32^, ^33^, ^34^ we tested whether the serum stimulation affects the AKT‐FOXO1‐RUNX2 axis in SaOS2. SaOS2 was shown in high RUNX2 and PPM1A expression levels relatively compared to other FOB, MG63 and U2OS osteoblastic cells (Figure S2A). Serum stimulation induced both phosphorylation at serine 473 of AKT and serine 256 of FOXO1A proteins in SaOS2 cells but reduced both mRNA and protein levels of FOXO1A and RUNX2 (Figure S2B). We also conducted transient co‐transfection with indicated gene constructs in control‐osteoprogenitor cells. As shown in Figure 3G, degradation of RUNX2 protein was shown in AKT expression but not in FOXO1 expression. In co‐transfection groups of RUNX2 and FOXO1A, RUNX2 protein was greater expressed than AKT or RUNX2 alone (Figure 3G, lanes 6 vs. 2 or 4), and dephosphorylation of FOXO1A protein was shown in exogenous PPM1A treatment (Figure 3G, lanes 6 vs. 7). Furthermore, the protein expressions of RUNX2 and FOXO1A were relatively stable in exogenous PPM1A treatment compared to vehicle (Figure 3H). Collectively, these results suggested that PPM1A upregulates RUNX2 expression via induction of dephosphorylation of FOXO1A protein.

## 4 DISCUSSION

In this study, the mRNA expressions of PPM1A and RUNX2 were significantly increased in facet joint tissues and osteoprogenitor cells of AS compared to the disease control. Exogenous PPM1A treatment also increased matrix mineralization of AS‐osteoprogenitor cells during osteoblasts differentiation. Upon exogenous PPM1A treatment, RUNX2 proteins were upregulated in both mRNA and protein of AS‐osteoprogenitor cells, but FOXO1A was increased only at the protein level. Mechanically, exogenous PPM1A treatment reduced the phosphorylated FOXO1A protein, thereby translocation of FOXO1A protein into the nucleus and an increase in RUNX2 expression. Taken together, these data findings indicate that the high expression of PPM1A increases matrix mineralization in AS via induction of the FOXO1A‐RUNX2 pathway.

Ankylosing spondylitis is chronic inflammatory arthritis that results in bone erosion or excessive bone formation.^35^ Bone erosion is a process where the bone surface is degraded or eroded by high osteoclast activity.^36^ On the other side, the bone‐forming process is controlled by bone morphogenetic proteins (BMPs) and the wingless family in osteoblasts.^37^, ^38^ In particular, BMPs are strongly associated with excessive osteoblast activity, abnormal bone formation, syndesmophyte formation and spinal ankylosis in AS.^39^, ^40^, ^41^, ^42^, ^43^, ^44^ Our study focused on bone formation in AS with elevated serum PPM1A levels.^16^ As a result, exogenous PPM1A treatment increased matrix mineralization in human AS‐osteoprogenitor cells suggesting PPM1A could be a serum biomarker for excessive osteoblast differentiation in AS.^18^ Functionally, PPM1A has been reported to dephosphorylate AMPK or transforming growth factor beta (TGFβ) and BMPs‐activated smad1/2/3/5 proteins.^10^, ^12^, ^14^ We confirmed using immunoblotting that exogenous PPM1A treatment did not affect TGFβ‐mediated phosphorylation of smad2/3 proteins in AS‐osteoprogenitor cells (data are not shown). We also observed that exogenous PPM1A treatment did not alter mRNA level of BMP genes in AS‐osteoprogenitor cells as well as phosphorylation of smad1/5 proteins by BMP6 (data are not shown). These results suggest that exogenous treatment with PPM1A exhibits different responses depending on type of cell in AS.

The functional role of FOXO1A is reported to regulate insulin signalling and glucose/lipid metabolism.^19^, ^45^ In general, FOXO1A is phosphorylated by kinase PI3K/AKT, while phosphorylated FOXO1A proteins were degraded by ubiquitin‐mediated proteasomes, resulting in the suppression of FOXO1A‐dependent target genes.^46^, ^47^ In our study, exogenous PPM1A stimulation did not affect phosphorylated AKT proteins (Figure 3B). We also showed a serum stimulation‐induced increase in the phosphorylation of FOXO1A and a decrease in total FOXO1A and RUNX2 proteins (Figure S2B). Therefore, we thought that FOXO1A proteins could be stabilized more in three phosphorylation mutants compared to the wild type in the presence of serum conditions. However, the FOXO1A mutants alone did not dramatically stabilize in FOXO1A proteins and upregulation of RUNX2 expression (data are not shown). In contrast, dephosphorylation of FOXO1A occurs in the nucleus, where FOXO1A binds to the promoter of the target gene to activate the transcription level. We also showed that FOXO1A overexpression upregulates RUNX2 proteins in 293 T cells, indicating that FOXO1A could exert positive RUNX2 transcript regulator (Figure 3G). Taken together, these results suggest that FOXO1A can control RUNX2 transcript level, and these relations were affected by serum.

Our study has several limitations. First, we did not confirm the knockdown or overexpression effects of the PPM1A gene because human primary osteoprogenitor cells are not ideal for transfection efficiency. Second, we did not identify the origin of exogenous PPM1A even though its expression was high in AS from our previous report. Therefore, further studies are needed to identify sources of PPM1A in AS. Third, although exogenous PPM1A treatment significantly inhibited the cell proliferation of AS‐osteoprogenitor cells (data are not shown), PPM1A‐responsible targets remain further studies.

In the present study, we demonstrated an actionable mechanism of PPM1A for the excessive matrix mineralization process of AS and proposed FOXO1A protein as a new target for PPM1A.

## AUTHOR CONTRIBUTIONS

Subin Weon involved in writing the original draft, methodology, data curation, formal analysis and visualization. Sungsin Jo involved in conceptualization, review and editing, funding acquisition, data curation, formal analysis and visualization. Bora Nam involved in data curation and formal analysis. Sung Hoon Choi and Ye‐Soo Park provided the resource. Yong‐Gil Kim involved in conceptualization, project administration, supervision, review and editing. Tae‐Hwan Kim involved in funding acquisition, project administration, supervision, review and editing.

## CONFLICT OF INTEREST STATEMENT

The authors declare no conflict of interest.

## Supporting information


AppendixS1

## Data Availability

The data that support the findings of this study are available from the corresponding author upon reasonable request.
