# Supplementary material for: Extracellular PPM1A promotes mineralization of osteoblasts differentiation in ankylosing spondylitis via the FOXO1A‐RUNX2 pathway
Source: J Cell Mol Med. 2023 Feb 9;27(5):650–8. doi: 10.1111/jcmm.17685 (PMC9983316; doi:10.1111/jcmm.17685)
Supplement: Supplementary file 1 — AppendixS1 [file JCMM-27-650-s001.docx]

Supplementary Table 1. Primer sequences used in RT-qPCR

| Gene | 5´-Forward-3´ | 5´-Reverse-3´ |
| --- | --- | --- |
| GAPDH | CAAGATCATCAGCAATGCC | CTGTGGTCATGAGTCCTTCC |
| PPM1A | ATGAGTCAGCACTGCCATAC | CCCACTATTACCTCCAGGAATC |
| FOXO1A | GGATGTGCATTCTATGGTGT | TTTCGGGATTGCTTATCTCA |
| RUNX2 | GTGGCCTTCAAGGTGGTAG | ACTCTTGCCTCGTCCACTC |
| OCN | ATGAGAGCCCTCACACTCCT | CTTGGACACAAAGGCTGCAC |
| OSX | TCTGCGGGACTCAACAACTC | TAGCATAGCCTGAGGTGGGT |
| DLX5 | TTCCAAGCTCCGTTCCAGAC | CCCCGTAGGGCTGTAGTAGT |

Supplementary Table 2. Antibodies used in the immunoblotting

| Antigen | Manufacturer | Species, Type | Catalog Number | Dilution |
| --- | --- | --- | --- | --- |
| PPM1A | NOVUS | Rabbit monoclonal | NBP1-32751 | 1:1000 |
| RUNX2 | Cell Signaling | Rabbit monoclonal | 12556 | 1:1000 |
| OCN | Abcam | Rabbit monoclonal | Ab133612 | 1:1000 |
| FOXO1A | Cell Signaling | Rabbit monoclonal | 2880 | 1:1000 |
| p-FOXO1A | Cell Signaling | Rabbit monoclonal | 9461 | 1:1000 |
| AKT | Cell Signaling | Rabbit monoclonal | 4691 | 1:1000 |
| p-AKT | Cell Signaling | Rabbit monoclonal | 4060 | 1:1000 |
| p-ERK | Cell Signaling | Rabbit monoclonal | 9101 | 1:1000 |
| p-p38 | Cell Signaling | Rabbit monoclonal | 9215 | 1:1000 |
| Active β-catenin | Cell Signaling | Rabbit monoclonal | 19807 | 1:1000 |
| HSP90 | BD Bioscience | Mouse monoclonal | 610419 | 1:1000 |
| Lamin B1 | Abam | Rabbit monoclonal | A1910 | 1:1000 |
| GAPDH | Cell Signaling | Rabbit monoclonal | 2118 | 1:10000 |
| HRP-conjugated goat-anti mouse | Jackson ImmunoResearch | Mouse | 115-035-003 | 1:2000 |
| HRP-conjugated goat-anti rabbit | Jackson ImmunoResearch | Rabbit | 111-035-003 | 1:2000 |

GAPDH: glyceraldehyde-3-phosphate dehydrogenase

PPM1A: protein phosphatase magnesium-dependent 1A

FOXO1A: forkhead box O1A

RUNX2: runt-related transcription factor 2

OCN: osteocalcin

OSX: osterix

DLX5: distal-less homeobox 5

AKT: protein kinase B

ERK: extracellular signal-regulated kinase

HSP90: heat shock protein 90

**Supplementary Figure legends**


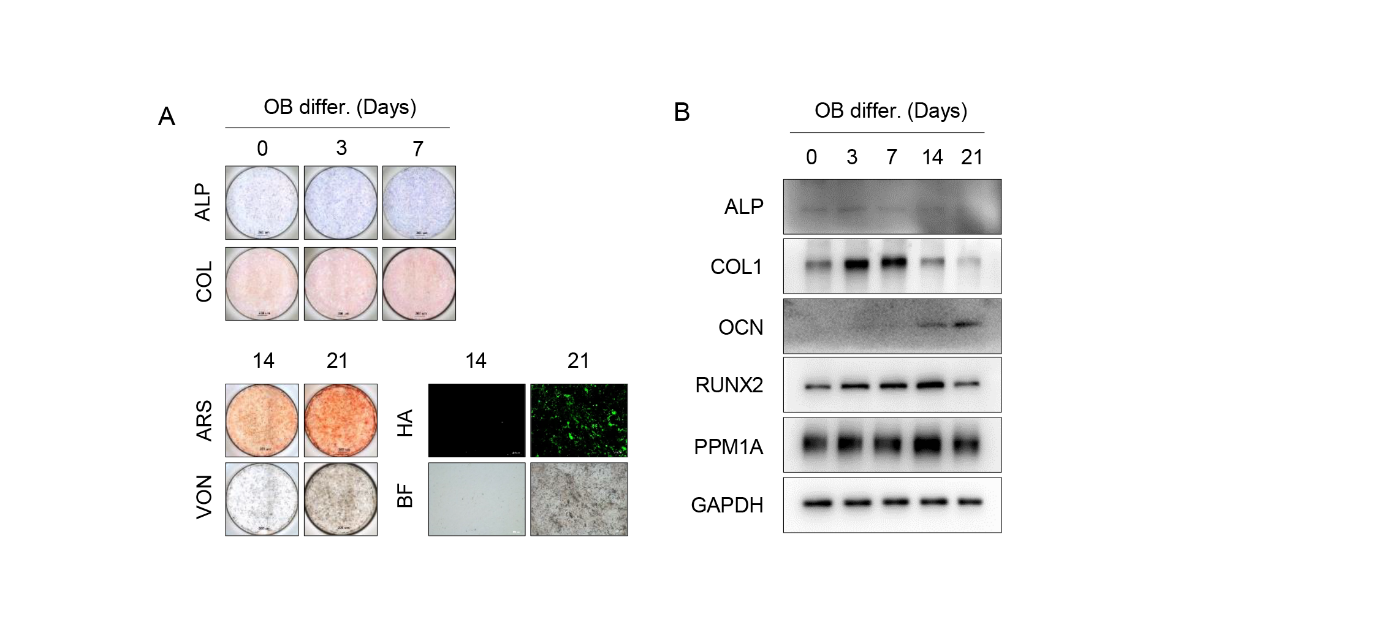


**Supplementary Figure 1. Expressions of RUNX2 and PPM1A were increased during osteoblast differentiation.** (A) Analysis of ALP, collagen, ARS, Von kossa and HA staining for control-osteoprogenitor cells during osteoblast differentiation at incubated days. Scale bar is 200 μm. (B) Verification of protein level using immunoblotting assay of osteogenic markers and PPM1A during osteoblast.


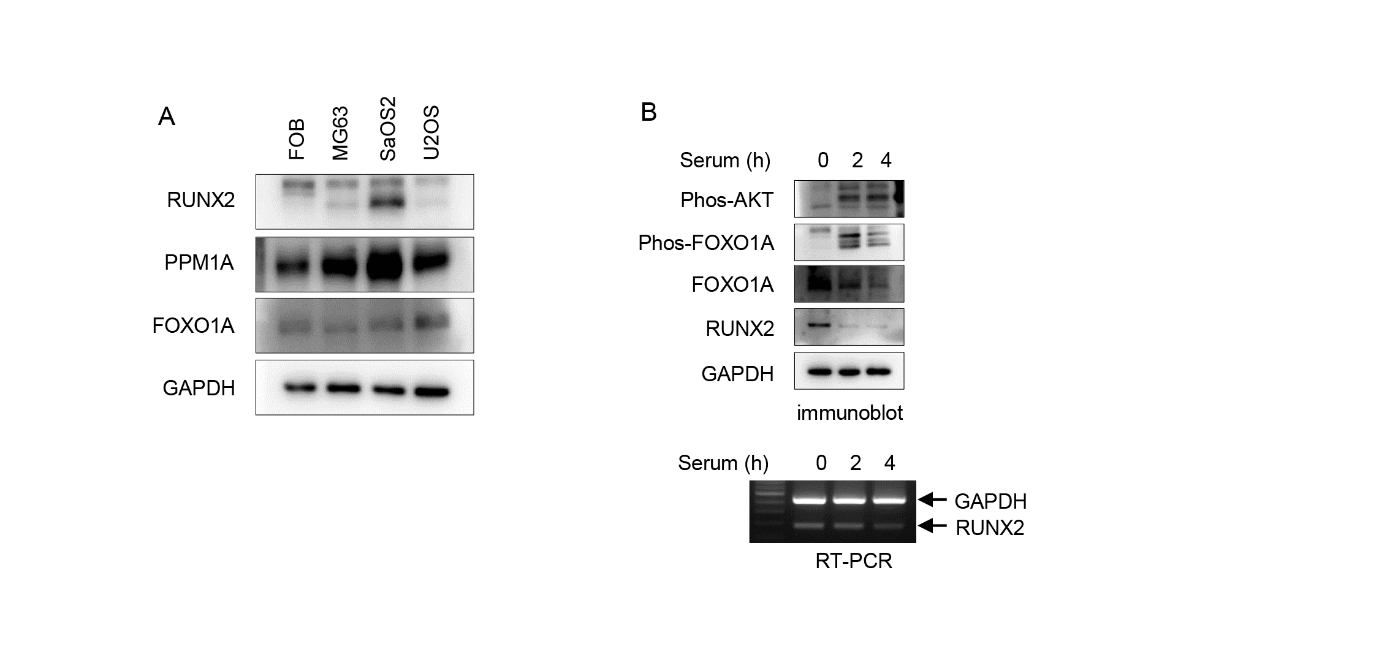


**Supplementary Figure 2. Serum stimulation reduced FOXO1A and RUNX2 by inducing phosphorylation of AKT and FOXO1A proteins.** (A) Protein expressions of RUNX2, PPM1A, and FOXO1A were compared in FOB, MG63, SaOS2, and U2OS cells. (B) SaOS2 cells were treated serum for 0 h, 2 h, and 4 h. Immunoblotting assay for protein level and RT-PCR for mRNA level were analyzed at each time points.
